# Editorial

**Published:** 1841-10-30

**Authors:** 


### THE MEDICAL EXAMINER.

PHILADELPHIA, OCT. 30, 1841.

### EPIDEMICS OF THE SUMMER AND AUTUMN OF 1841.

Taking the season together, it has been unhealthy throughout the United States. The eastern states with their usual immunity against the diseases of warm climates, have almost escaped, but throughout the greater part of the United States the summer diseases have been unusually prevalent, and have extended to localities which are ordinarily exempt from their visitations, while other places in which the malarias are endemic have suffered from them to a severe and often fatal degree.

It is rare for remittents to appear in the thickly inhabited parts of the city of Philadelphia, but such cases have not been at all uncommon during the present season. It is true, that in many instances the disease was evidently contracted in the country, but such an origin was not apparent in every case—a strong proof of the extension and prevalence of the series. The epidemics have, as usual, varied during different parts of the season, in different parts of the country; in the hilly districts dysentery prevailed throughout the summer and early autumn; in the flat country, and near rivers, dysentery appeared in the months of July and August, but became much less common in September and October, when fevers were more rife. But there was in many cases a close connection between the two classes of disease, one passing as it were into the other, sometimes appearing simultaneously in the same patient, at other times different members of the same family were taken down about the same period with dysentery and fever, so that there was probably one cause producing different effects. The epidemic which visited a large portion of Chester county, in this State, was almost confined to dysentery: the face of the country is free from the usual causes of malaria, and the district is in general remarkably healthy, but, during the present season, a severe, and often a malignant form of dysentery was prevalent, causing many deaths. It assumed the malignant type very early, with great prostration of strength, reddish fœtid stools, and other signs of mortification of the mucous membrane.

The remittents and intermittents of the vicinity of Philadelphia appeared later in the season than usual; few cases being seen before the month of September. The two forms were, as usual, convertible one into the other, but the proportion of remittents, compared with intermittents, was larger than usual, and the remissions were less marked, so that the fevers were the pseudo-continued, resembling in some respects typhoid fevers, but differing in the absence of some of the characteristic symptoms, such as the mode of invasion, the spots on the abdomen, and the progress of the disease. Still, as the cerebral symptoms were well marked, and in a great proportion of cases attended with stupor and muttering delirium, the fever would have been called typhoid by those who do not restrict the term to the single form of disease to which it should be given; in other words, some of the prominent symptoms of typhoid fever were closely imitated in many of the remittents of this year. This form of remittent, as is generally the case, has borne quinine badly; in fact, it belonged to those cases in which cinchona was not the remedy, and was only tolerated, like other tonics, after the conclusion of the fever during the feebleness of convalescence. The best treatment was bleeding when the reaction was high, but generally merely local depletion, mercurial, and other purgatives, cold applications to the head, and the usual diaphoretic drinks. The other form of remittents was much more distinctly paroxysmal, the fever declined or ceased during the remissions, and the paroxysms in some cases were mild, and in others were accompanied by profuse stupor and prostration; in other words, assumed the type known on the continent as pernicious intermittent, and in this country as congestive fever. The free employment of blisters and stimulating applications to the skin and enemata during the paroxysm, with the liberal use of quinine in the remissions, was absolutely necessary.

These forms were almost the only ones met with; there were, we believe, no cases of the prolonged remittent with jaundice and intense cerebral disturbance, and but few of the mild form which prevailed here in the year 1839, in which the fever was extremely slight, the cerebral functions almost intact, and the disease terminated quickly in recovery, or subsided into the usual intermittent type, which was then readily cured by quinine. The forms, however, of one continual intermittent are so irregular and diversified, that the modifications of the disease in different parts of the country were probably extremely numerous.

In a few cases of great severity the fever was complicated, and, at first, masked by pectoral inflammations. In these cases the patient had been exposed to miasmatic influences, and afterwards to cold, wet, or other ordinary causes of inflammations. As soon as these had subsided, the autumnal fever was developed with unusual severity.

The anxiety which we naturally feel to diffuse as much information as possible as to the epidemic diseases of the country, induces us to request our contributors to furnish any facts of interest which may come under their notice, in relation to the diseases of this year. They may render essential services by their contributions.

---

### MEDICAL SCHOOL OF PHILADELPHIA.

The winter session of the great medical school of Philadelphia has commenced, and the introductory addresses of the professors of the three collegiate institutions which grace our city are in full progress. It becomes our duty, as journalists, to note, and perhaps occasionally to comment upon the events occurring during the term which are calculated to awaken the attention or effect the interests of the whole profession, at present or prospectively, and we shall endeavour to perform the task in a perfectly impartial manner.

This journal is the organ of no party in medical politics, nor is it bound by any merely local considerations, but is designed to forward the progress of professional knowledge, and assist in elevating the professional character in an honourable and courteous manner, and on principles purely cosmopolitan. If in noticing, and possibly uttering an occasional criticism upon the proceeding of the very numerous medical institutions of the country, an undue share of attention should appear to be given to the claims of those immediately around us, this will not be the result of any narrow calculation or partial views, but may be safely attributed to our local position alone.

Within the last fifteen years the means of acquiring information in medicine and the collateral sciences in Philadelphia have been vastly increased, partly by the multiplication of charitable and other foundations, partly by improvements in the organization of those previously existing, and partly by the establishment of more numerous private lectureships or combinations of teachers in the several departments. But although the resources for instruction have been more fully developed here than in any other American capital, there is still room for immense improvement in this respect, and the spirit is now fully awake which must bring about its accomplishmeut.

To those who visit our city for the sole purpose of obtaining a degree—whose residence among us is restricted to the few short months of the established curriculum, a knowledge of these resources, though important, is not so absolutely essential as to those who possess the means and disposition to reap every advantage afforded them without so close an attention to the amount of time expended in acquisition. The gentlemen who are placed in the closest relations with the medical class have observed with gratification that, while it maintains its numbers very steadily, there is an obvious annual improvement in its material, and there is just ground for regret that so few of the numbers composing it are made aware of the value of the information obtainable at other seasons and from other sources than the collegiate lectures and the winter session. We shall endeavour hereafter slowly to extend the knowledge of these advantages within the range of our circulation, so that the preceptor at a distance, and the pupil even before his arrival, may form a better idea of the extent and boundaries of the field on which the latter is about to enter. To accomplish this purpose suddenly would be impossible—so numerous are the institutions and combinations *that are, or should be made tributary* to the medical school of Philadelphia.

An honourable rivalry between similar establishments exists amongst us, not only in the case of the colleges, but in other instances also. Long may it continue! As long as it can be conducted on terms of mutual good feeling, and in such a manner as to elevate, instead of depressing the dignity of the profession and the quality of medical instruction! The time has arrrived when the only mode in which any particular school can gain and preserve preeminence is by rendering itself superior in merit. It is no longer possible to break down an institution that is governed upon correct and enlarged principles, by drawing off or striking from the list of its supporters a few distinguished names. The importance of individuals is diminished as the mass of talent and learning in the profession increases with the growth of the country, and all such attempts must fail. It has become equally impossible to secure and retain success by a loud appeal to the prejudices of the common public, and by displays which captivate for the moment, unbacked by solid learning.

The school that pursues an honourable, gentlemanly, professional course among its competitors, will always be supported, if it possess the means of instruction in hospitals, libraries, and cabinets, the ability to teach, in able professors and proper subsidiary aid, and a disposition to expand with the progress of the age. Philadelphia continues to enjoy these advantages. Should she, through folly, lose them, we would be among the last to raise a hand to save her from the consequences. The same principles must also control the fate of the contending rivals within her bosom. At present, the medical atmosphere is, perhaps, unusually clear.

The class, so far as we can judge from the usual assemblage at the introductory lectures delivered at the different colleges, fully maintains its pre-eminence in numbers, in the face of ever active competition, and the disturbance of the currency. Its positive amount, and the ratio of its divisions among rival foundations, we have, at present, no authentic means of estimating. When ascertained, it will be duly reported.

We have snatched from the time necessarily devoted to other pressing engagements, a few moments only, for the purpose of hearing portions of at least one of the addresses delivered at each of the collegiate establishments of our school ; and in noticing them, the most correct method of avoiding all suspicion of party bias, will be to observe an alphabetical order.

*Lecture of Dr. Bird.*—Although the magical number *six* no longer controls the appointment of a medical faculty, and the school of Philadelphia now offers a band of collegiate lecturers exceeding twenty—although the extreme right of the conservative party in medical politics sometimes declares that "it has become almost a matter of distinction *not to be a professor*"—the advent of a new appointment to ministerial office in the collegiate branch of medical instruction is, and ought to be regarded as an important event in professional annals. It effects the progress of the science not in a temporary, but in a permanent manner ; for the seeds of instruction collected in early life preserve more accurately the peculiarities of the soil in which their parents grew, and extend those accidental influences to posterity more surely and powerfully than the tree that is transported from one region to another. The choice of a good or a bad teacher in medicine in a school of extended reputation—which that of Philadelphia ever has been—produces effects in future times upon the advancement or deterioration of the science and its institutions, after the name of the individual is almost forgotten.

At the Pennsylvania College, last evening, we had the good fortune to hear the latter half of the "maiden address" of Dr. Bird, and must plead guilty to an agreeable disappointment. It is almost impossible, in many cases, to resist entirely the effects of popular prejudice, even when our own experience has proved its fallacy, and we could not entirely resist the fear that the application of acknowledged genius to the lighter class of literary labours might have destroyed the habit of close observation, and the respect for inductive reasoning so necessary to the labourer in his present abstract field of duty. That this is not the case, is sufficiently certified by the character of the address alone. Classical, we knew it would be, from some knowledge of his earlier tastes and habit of thinking; but we were not prepared for the terseness, the vigor of style, and the freedom from exuberant ornament which carried conviction to the mind of every one that he was listening to a scholar, not liable to be led from the direct path of research, to sport among the flowers of the imagination blooming by the way-side. That he *saw* them, appreciated their beauty, and was prepared to lighten the tedium of the journey by an occasional reference, was sufficiently evident, but he spent no time in weaving garlands. There was no unnecessary flattery of others or the class—no leaning upon great names to buoy himself up, as though conscious of the danger of sinking in the current—no catering to a vitiated taste at the expense of the dignity of his subject and position. We speak from what we heard, but feel no fear that the author of the latter portion of the lecture could have rendered these acknowledgements unjust by any previous remark.

There was but a single point which seemed to provoke criticism, and this *appeared* the result of a habit of preferring the study of settled facts and incontrovertible conclusions to the strict analysis of the relative merits of hypotheses none of which are entirely well founded, though embracing many truths. It struck us, at the moment, that he might prove—*very slightly*—ultra-eclectic: and even this is rather a virtue than a fault in a teacher of the Materia Medica. But we must protest against ranking the vague dream of Homœopathy among the medical *theories*. It does not rise to the dignity of a hypothesis—for it is not placed in such order as to be even worthy of inquiry. It stands by the side of the *miscalled science* of Mesmerism, in which a number of most curious and important physical facts, well worthy of investigation, are attempted to be associated on certain *miscalled principles* drawn from regions *beyond the land of dreams*. So far as we have been able to collate and rigidly condense the fundamental dogmas of that which is not a doctrine, because it is unteachable, they are as follows:

1st. Effects cannot be distinguished from their causes!

2d. The half, *when properly triturated*, is greater than the whole!

3d. The best way to cure a disease is to make it a little worse!

In manner, Dr. Bird is agreeable and impressive. Some slight undue rapidity of utterance, a little occasional falling of a voice not lacking in power until it became inaudible at the rear of the room, and the absence of free movement, were probably due to a trace of the natural embarrassment which has often caused us to *hug the desk* much more affectionately on far less trying occasions. In short—the lecture was any thing but *a flourish of trumpets*. Let us now look in upon friends of longer standing in the ranks of medical teaching.

*Lecture of Dr. Dunglison.*—The principal characteristic of this discourse, delivered on Monday evening at Jefferson Medical College, was good nature. It would be supererogatory to speak of the style of this well known writer and teacher; his professional merits and peculiarities are as wide-spread as the profession in his native and adopted countries. The former rank deservedly high;—the latter are proper to his temperament. We could not but admire the care, without the appearance of care, with which he so adroitly managed to avoid every expression which could be misconstrued into personality or severity, placed as he is, in the midst of rival institutions, the one the parent, and the other the offspring of that with which he is at present connected.

Dr. Dunglison has been deeply involved in the changes and revolutions of the profession for a good many years, and has passed through many trying scenes, making fewer enemies than most men, under similar circumstances, would have gathered around them; and this is no mean praise.

The only points in the lecture which call for remark are the statements of the resources of the college in the way of cabinets, &c., the compliments paid to the operation of tenotomy, and those addressed to his colleagues.

It has been said, in the earlier part of this article, that the numerical increase of the profession was rapidly rendering individuals less important in science, and that it was no longer possible to break down the standing of *a really well governed* school by removing particular props, on which, for the moment, its safety may seem to depend. This remark was made in a national sense: the present assertion is true, perhaps, in relation to our own city only. Such has been the long continued devotion to medical and natural science generally in Philadelphia, that cabinets and resources for the formation of cabinets are multiplied beyond the demands of mere elementary teaching. Such material is accessible within reasonable time, and by means of reasonable exertion on the part of any body of teachers who are disposed to meet the requisite expense and trouble. No faculty in this city could be broken down or permanently injured by the total destruction of its cabinets, small as these generally are in comparison with our resources. We thought the semi-deprecatory remarks on this head unnecessary, but perhaps they may have been proper and requisite before an audience of students, many of whom are among us for the first time.

Dr. Dunglison ranked the operation of tenotomy as among the most important of modern surgical triumphs. This is elevating it far above its proper position. Though our pages bear a series of articles on this subject tending to condemn the indiscriminate resort to this kind of operation, the war is not against the operation itself, exceedingly simple as it is, but against the spirit of hyper-operating which has become, of late, so fashionable as to disgrace the profession. We feel perfectly confident that his colleague, the Professor of Surgery, will agree with us in this opinion, and it is only the influence of the undue praise of a proceeding so often resorted to without sufficient cause, upon the minds of the less mature and more ambitious spirits, that is to be deprecated. That tenotomy is not unfrequently proper in various deformities, no one who understands the principles of surgery will venture to deny.

The compliments paid to his several coadjutors, by Dr. D., were well turned and appropriate. One of the richest of these fell upon the Professor of the Institutes and Practice, who was the first to intimate, in this country, the processes of Farraday and Thillorier on the liquifaction and solidification of carbonic acid gas.

*Dr. Wood's Lecture.*—At the University of Pennsylvania, the only discourse which we have been able to attend, was that of the professor of the materia medica, and even of this the conclusion only. It were supererogatory to say that the style was lucid and classically chaste. The labours of his pen are always thus. He was descanting at the moment upon the various and impudent pretensions of quackery in a manly, dignified, and decided manner —not casting away indignation upon that which is beneath it. Upon the subject of the so styled animal magnetism, he took the proper ground, acknowledging the fact of the cataleptic condition into which certain classes of patients are thrown by those who practice certain curious manipulations, and stating the truth, which must be perceived by all who are sufficiently philosophical to reason correctly, that the physical influence of the nervous system of one individual over that of another, is not *absolutely beyond the range of physical possibility*, he condemned the spirit which would ridicule those who are disposed to investigate the facts in relation to this subject which furnish legitimate data for the furtherance of inductive philosophy while he repudiated the idle nonsense which loads these facts with miraculous circumstances.

As this lecture should be, and probably will be published, we will close this article with the remark that the only possible differences of opinion on animal magnetism capable of discussion between really sound reasoners, will turn upon the extent and nature of the nervous functions, and their appropriate stimuli. It is a question purely physiological, and therefore physical—difficult, it is true, but solvable. In future numbers notice will be taken of the experiments now in progress, and of the peculiar views of some of our observers on this subject which so many fly from with idle dread.

